# The ultra-sonication of minerals in swine feed

**DOI:** 10.1186/s40104-015-0030-3

**Published:** 2015-08-04

**Authors:** Mayra A. D. Saleh, Pedro M. Padilha, Lucélia Hauptli, Dirlei A. Berto

**Affiliations:** Department of Animal Production, Lageado Farm, Faculty of Animal Science and Veterinary Medicine, UNESP – São Paulo State University, Botucatu Campus, 18618-000 Botucatu, São Paulo Brazil; Department of Chemistry and Biochemistry, Institute of Biosciences, UNESP – São Paulo State University, Botucatu Campus, Rubião Junior District, 18618-970 Botucatu, São Paulo Brazil; UFSC – Federal University of Santa Catarina, Center for Agricultural Sciences, 88040-900 Florianópolis, Brazil

**Keywords:** Digestibility assay, Green analytical chemistry, Macronutrients, Piglet, Ultrasound-assisted extraction

## Abstract

A sample preparation method based on ultrasound assisted-extraction (UAE) of Ca, Mg and P from swine feed has been described. The experiment was performed to cover the variables influencing the sonication process and, the method validation using standard reference material. Final solutions obtained upon sonication were analyzed by flame atomic absorption spectrometry (for Ca and Mg) and by UV–vis spectrophotometry (for P). The best conditions for metal extraction were as follows: sample mass: 100 mg in 20 mL 0.10 mol/L HCl, a particle size: <60 μm, sonication time: 5 cycles of 10 s and ultrasound power: 102 W. The UAE method was applied in digestibility assays in different piglet feeds and their results showed that it is highly comparable (*P > 0.05*) to the other methods used for such purposes, as block digestion, and offered a Ca, Mg and P method of quantification limit of 10.6, 12.4 and 14 mg/kg, respectively. The major advantages of the UAE method compared to other methods are the high treatment rate, low reagent usage in the extracts and, it does not generate toxic residues that might negatively affect human health and the environment, accompanied by good precision and accuracy.

## Introduction

The animal feed industry adopted the strategy of using more digestible ingredients in the formulations to minimize the inclusion of minerals and maximize the efficiency of nutrient used by the animal while minimizing the negative effects of weaning, the critical period in pig farms, ensuring the performance desirable at this stage of the piglet production, and even contributing to the reduction of environmental impact caused by its waste [1, 2].

However, the use of external markers (Cr_2_O_3_) for the estimation of the apparent digestibility coefficient of nutrients metabolized by the animal is problematic due to the expense of the large amounts of it administered to obtain analytical results and appropriate variations in the research results, mainly due to analytical variability [3–5]. Moreover, the quantification of Cr_2_O_3_ in feed and feces presents difficulties in sample preparation procedures, which plays a key role in determining the success of subsequent analysis.

The mineralization of samples is performed by slow heating in digestion blocks, using a nitric-perchloric mixture which involves the use of high temperatures or corrosive reagents, usually under pressure, which is a tedious and time-consuming treatment using concentrated and harmful reagents that demand very stringent safety conditions. The resulting extracts are acid solutions containing the inorganic nutrients and the dichromate ions (Cr_2_O_7_^2−^), a highly toxic species that negatively affects human health and contaminates the environment [6].

Because of the negative effects of this procedure, the development of new methodologies that allow the safe quantification of inorganic nutrients in studies of animal nutrition has become crucial, mainly due to the importance of calcium, magnesium and phosphorus in biochemical processes. The use of ultrasound-assisted extraction (UAE) for the separation of nutrients from biological samples is presented as promising, mainly because the energy released during acoustic cavitation offers excellent prospects for sample preparation [7, 8], such as low volumes of solvent needed, reduction in analytical time, and the possibility of developing fully automated methods, allowing the elimination of acid mineralization [9–13], and hence contribute to the achievement of the green principles in analytical chemistry.

Due to the above, this study evaluated the efficiency of ultrasound-assisted extraction as an alternative to acid mineralization method in extracting calcium, magnesium and phosphorus from piglet feed followed by determination of the Ca and Mg concentrations by flame atomic absorption spectrometry (FAAS) and the P concentrations by ultraviolet–visible spectrophotometry (UV–vis) and, the subsequent estimation of the apparent digestibility coefficient of these nutrients.

## Materials and methods

The research project was conducted at UNESP (São Paulo State University) at the Faculty of Veterinary Medicine and Animal Science, Botucatu campus, with the approval by the Animal Ethics Committee from this institution (protocol number 114/2006 – CEUA) and, in accordance with the directive 86/609/EEC.

### Housing, animals, diets and experimental design

The digestibility experiments were conducted in the experimental facilities of São Paulo State University (UNESP) at Botucatu. A total of 90 crossbred commercial lineage piglets were weaned at 21-days-old and exhibited an average weight of 5.42 ± 0.55 kg.

The animals were allotted into 3 treatments groups (1: soybean oil, 2: palma oil and 3: blend of palma oil, dry skim milk and maltodextrin) following a randomized complete block design based on their initial body weight and sex with 10 replicates and 3 piglets per pen. The animals were housed in a nursery facility with a ceiling height of 3.5 m and lateral curtains and, suspended in metal pens with an area of 1.70 m^2^ and campanula heating. The pens had a partially slatted plastic flooring and a compact concrete floor under the campanula. There was a water layer under the pens that was replaced once or twice a week depending on the volume of waste produced.

The diets were formulated in accordance with Rostagno’s recommendations (Table 1) [14]. All pigs were visually evaluated at weaning to check their health status, and only animals appraised as healthy were used in this study. There was one nipple drinker and one feeder per pen, and feed and water were provided *ad libitum*.

**Table 1:** Composition of experimental diets fed young pigs

Ingredients	Composition, %
Ground corn	52.265
Soybean meal	20.000
Gluten	2.850
Yeast	4.000
Spray-dried blood meal	1.500
Blend^a^	*
Fumaric acid	0.600
Dry skim milk	*
Maltodextrin	*
Soybean oil	*
Palma oil	*
Limestone	0.750
Dicalcium phosphate	2.000
Salt	0.300
L-Lysine HCl (78.4 %)	0.620
DL-Methionine (99 %)	0.110
L-Threonine (98.5 %)	0.270
L-Tryptophan (98 %)	0.045
ZnO (73 %)	0.340
Antioxidant^1^	0.020
Sweetener^2^	0.020
Choline chloride	0.040
Vitamin premix^3^	0.100
Mineral premix^4^	0.100
Chemical Composition (Calculated Values)	
Metabolizable energy, kcal/kg	3,400
Protein, %	19.50
Lysine, %	1.50
Methionine, %	0.42
Threonine, %	1.03
Tryptophan, %	0.26
Calcium, %	0.88
Phosphorus, %	0.68

^a^Blend composition: 30.30 % palma oil; 10.40 % dry skim milk; 59.30 % maltodextrin

^1^Butylated hydroxy toluene; ^2^Sodium saccharin

^3^Supplied per kg of diet: 9,000 UI vitamin A; 2,250 UI vitamin D_3_; 22.5 mg vitamin E; 22.5 mg vitamin K_3_; 2.03 mg vitamin B_1_; 6 mg vitamin B_2_; 3 mg vitamin B_6_; 30 μg vitamin B_12_; 0.9 mg folic acid; 14.03 mg pantothenic acid; 30 mg niacin; 0.12 mg biotin; 400 mg de choline; ^4^Supplied per kg of diet: 100 mg Fe; 10 mg Cu; 40 mg Mn; 100 mg Zn; 1 mg Co; 1.5 mg I

*Treatment 1: 1.040 % dry skim milk, 10.000 % maltodextrin, 3.030 % soybean oil

*Treatment 2: 1.040 % dry skim milk, 10.000 % maltodextrin, 3.030 % palma oil

*Treatment 3: 4.070 % maltodextrin, 10 % blend

Partial collection of feces using chromium oxide 0.1 % as an external marker of pre-starter diets was performed in the second experimental week to determine the apparent digestibility coefficients (ADC) using the equation 1, according to [15].

1 $$ \mathrm{A}\mathrm{D}\mathrm{C}=100-\left[100\times \left(\frac{\%{Cr}_2{\mathrm{O}}_3\mathrm{r}}{\%{Cr}_2{\mathrm{O}}_3\mathrm{f}}\right)\times \left(\frac{\%\mathrm{N}\mathrm{f}}{\%\mathrm{N}\mathrm{f}}\right)\right] $$

where:

- ADC = apparent digestibility coefficient,
- %Cr_2_O_3_r = percentage of chromium oxide in the feed,
- %Cr_2_O_3_f = percentage of chromium oxide in the feces,
- %Nr = percentage of nutrients in the feed,
- %Nf = percentage of nutrients in the feces

The feed marked with the chromium oxide was provided to piglets starting on the 7th day of the trial period, and the fecal samples were collected between day 11 and day 14 at 8:00 h and 16:00 h, identified and stored frozen (−20 °C) in plastic containers containing 1 kg of samples (multiple samples of three piglets per pen during the four-days collection period).

At the end of the experiment, samples of feed and feces from each pen were dehydrated in circulating air drying oven at 45 °C for 48 h and milled in a knives-type mill (0.595 mm). Subsequently, a mass of approximately 100.0 g of sample was sent to the laboratory for the analytical procedures.

### Reagents and solutions

Ultrapure water (18.2 MΩ/cm) obtained from the PURELAB Ultra Ionic Elga system (Technology Drive Lowell, MA, USA), and spectroscopic grade nitric and hydrochloric acid (Darmstadt, Germany) were used. Stock solutions of the analytes and concomitants were prepared by dilution of Merck tritisol standards (Darmstadt, Germany). The remaining solutions including the extracting solutions were prepared from analytical grade reagents. All of the solutions were stored in polypropylene flasks.

All of the stock flasks of samples and standard solutions, glassware and accessories of the atomic absorption spectrometer (aspiration and spray system) and the UV–vis spectrophotometer were cleaned in nitric acid 10 % v/v for 24 h, then rinsed in ultrapure water and dried with jets of pure air before use.

### Sample preparation

Piglet feed and feces samples were cryogenically milled. To this end, a mass of approximately 1.00 g of sample was placed together with a magnetic bar into a polycarbonate jar, which was then closed and immersed in liquid nitrogen. The impact between the sample and the magnetic bar subjected to an oscillating magnetic field (20 impacts/s) pulverized the sample. The sample milling procedure consisted of an initial stage of 2 min of pre-freezing, 1 min for pulverization, and 1 min of freezing, followed by a second stage consisting of two cycles with two pulverization and freezing steps, for a total time of 8 min. This procedure produced particle size less than 60 μm [16, 17].

### Extraction of the analytes

#### Acid mineralization (Ca, Mg and Cr)

After the drying and milling stage, part of the samples was mineralized in digestion blocks. To this end, masses of 100.0 mg of the cryogenically milled samples were transferred directly to 100 mL borosilicate glass tubes (Kjeldahl type), to which were added 3.0 mL of nitric acid 65 % m/m (HNO_3_ in pure form) was added at 100 °C initially. After complete oxidation of the organic matter, 1.0 mL of 70 % m/m perchloric acid (HClO_4_ in pure form) was added at 200 °C. The acid extracts obtained were diluted to 50 mL with ultrapure water*.*

#### Acid mineralization (P)

For the UV–vis analysis, samples were digested by acid mineralization in which 5.0 mL of nitric acid 65 % m/m (HNO_3_ in pure form) were poured to the homogenized sample (0.1 ± 0.0001 g) and it was left for 24 h into the Kjeldahl flasks. Afterwards, 2.0 mL of hydrogen peroxide 30 % m/m (H_2_O_2_ in pure form) were added to the mixture, heated for 60 min at 100 °C, and for additional 120 min at 200 °C, resulting in a clear solution which was transferred into a 50 mL volumetric flask and filled to the mark.

#### Ultrasound-assisted extraction

After the drying and milling stage, approximately 100.0 mg of sample and 20 mL of 0.10 mol/L HCl solution were transferred to 30 mL teflon flasks. The mixture was then subjected to ultrasonic shaking to extract the analytes. This procedure allowed the evaluation of different sonication times and ultrasonic powers in the analyte extraction process. The acid extracts obtained were separated from the remaining solid phase by centrifugation (3,500 rpm at 24 °C for 5 min).

#### Accuracy check for the UAE method

The quantification limit (QL) of the analytical curve was calculated in accordance to IUPAC procedures (QL = 10 s_B_/S, in which s_B_ is the standard deviation of 20 consecutive measurements of the blank solution and S is the slope of the respective curves) [18]. The method of quantification limit (MQL) was determined following to [19].

#### Application of the proposed method

Based on the values of the concentration of the nutrients and the percentage of chromium oxide determined in the samples under study, a calculation was made to estimate the apparent digestibility coefficients (ADC) of these nutrients permitting the comparison of both extraction methods.

#### Apparatus

The piglet (feed and feces) samples were dried in a MARCONI model MA-035 (Piracicaba, Brazil) circulating air drying oven, milled in a freezer cryogenic impact mill SPEX model 6750 (Metuchen, EUA) and mineralized in digestion blocks MARCONI model MA-447/6/100 (Piracicaba, Brazil).

An ultrasonic cell disrupter (horn-type ultrasonic probe) UNIQUE model USC-DC (Campinas, Brazil) equipped with a 3 mm titanium probe and operated at frequency of 20 kHz was used in the ultrasound-assisted extraction of the analytes. An ultracentrifuge model Bioagency Bio-Spin-320R (São Paulo, Brazil) was used to separate the ultrasound extracts from the remaining solid phase.

Calcium and magnesium were determined by using an atomic absorption spectrometer SHIMADZU AA-6800 (Tokyo, Japan) equipped with a background absorption correction with a deuterium lamp and self-reverse (SR) system. Phosphorus was measured using UV/Vis spectrophotometer Thermo Spectronic model Genesys 6 (Madison, EUA) equipped with a xenon lamp and an automatic six-cell changer with 1 cm quartz cells.

### Analytical procedures

#### Analytical curves

Standard mixed aqueous solutions of Ca, Mg and Cr were prepared from the dilution of Tritisol standards (Merck) containing 1,000 mg/L of the analytes in a medium of HCl 0.10 mol/L and 10 g/L of La^3+^ (used only for Ca and Mg standards). For each one, a blank was prepared containing all the components of the standard solutions except the analyte of interest.

For calibration, five standard solutions of phosphorus were prepared from the dilution of monopotassium phosphate (KH_2_PO_4_) 1.25 mmol/L and by the addition of 10.0 mL of the molybdate–vanadate reagent. The obtained solutions were left for 15 min to ensure the colour development and the absorbance signals were measured in acidic medium against the blank at λ = 420 nm with three replicates. The HCl solution 0.10 mol/L, after an addition of the molybdate–vanadate reagent was used as the blank for determination of phosphorus.

The optimal ranges of concentration of the analytical curves were as it follows: Ca: 0.5 to 4.0 mg/L; Mg: 0.1 to 0.5 mg/L; P: 1.55 to 9.29 mg/L and Cr: 1.0 to 8.0 mg/L. The equations for the calibration graphs obtained were as it follows: Ca: Abs = 0.05824 C_Ca_ + 0.001323, r = 0.9999; Mg: Abs = 1.4899 C_Mg_ + 0.03365, r = 0.9996; P: Abs = 0.0528 x C_P_ + 0,00494, r = 0.9999 and, Cr: Abs = 0.0273 C_Cr_ + 0.0101, r = 0.9993.

#### FAAS calibration

The operational conditions employed in the determinations of Ca, Mg and Cr were:

Ca λ = 422.7 nm, spectra resolution: 0.5 nm, lamp current: 10 mA, flame stoichiometry: oxidizer, flame type: air-acetylene and gas flow 2.0 L/min; Mg λ = 285.2 nm, spectra resolution: 0.5 nm, lamp current: 10 mA, flame stoichiometry: oxidizer, flame type: air-acetylene and gas flow 1.8 L/min and Cr λ = 357.9 nm, spectra resolution: 0.5 nm, lamp current: 10 mA (low) and 400 mA (high), flame stoichiometry: slightly reducer, flame type: air-acetylene and gas flow 1.8 L/min.

The determination of chromium was necessary to calculate the chromium oxide content present in feed and feces for the subsequent estimation of the apparent digestibility coefficient of Ca, Mg and P. The absorbance signals were measured in the peak area, according to Welz (1992) [20].

#### UAE calibration

The efficiency of the method in the analyte extraction was evaluated by varying the extraction time and ultrasonic power. This testing values were established by prior studies which utilized similar matrices and have found satisfactory extraction effects [21, 22]. The available time setting range was in 10 s interval up to 60 s. Tests at lower levels of time and powers of sonication than the minimum used here were not possible, due to operational specification settings.

Therefore, the minimum configurable time of the ultrasound digital timer started with 10 s. The sonication time testing was carried out varying the time scale by 10 units at time (10, 20, 30, 40 and 50 s) and evaluating the cycles of sonication time (1, 2, 3, 4 and 5 cycles). Initially, the tests were performed fixing the sonication time at 1 cycle of 10 s and varying the sonication power from 20 to 70 % and evaluating the absorbance signals of each analyte. Thereafter, the remaining combination were carried out (e.g. 1 cycle of 20 s and the evaluation from 20 to 70 % range of the sonication amplitude).

Concerning the levels of sonication power, the allowed power setting was from 20 to 90 % of total power operation (340 W). The range of 20 to 70 % of total power operation of the ultrasound was chosen because levels above 70 % caused an overheating of it, although the equipment was calibrated, and it was given proper maintenance.

The effect of the hydrochloric acid concentration on the process of extracting the analytes from the samples studied was also considered. The absorbance signals increased up to a concentration of 0.10 mol/L of acid, remaining constant in the 0.10 – 1.00 mol/L range. Thus, in all the remaining experiments, a 0.10 mol/L hydrochloric acid was used as extracting solution. The fixed volume adopted (20 mL) has been chosen due to prior literature results which achieved easily homogeneized liquid phase of the solution and lead to higher analyte extraction in acidic medium when ultrasonic probes were used [23–26].

### Statistical analysis

All experimental data were analyzed in accordance with the general linear model procedure established by the Statistics Analysis System Institutes [27]. The Tukey’s test was used to demonstrate statistical differences between groups. The pen of three pigs was considered to be the experimental unit for statistical analyses in this experiment. The variability of the data was expressed as the standard deviation and a probability level of (*P < 0.05*) was considered to be statistically significant*.*

## Results and discussion

**Fig. 1:**
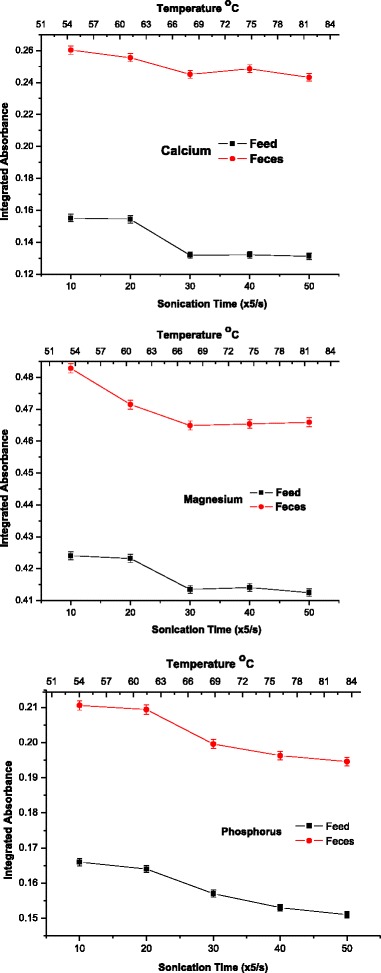
Influence of sonication time on the process of calcium, magnesium and phosphorus extraction from samples of piglet feed and feces. Experimental conditions: power 102 W; extracting solution hydrochloric acid 0.10 mol/L

During the process of extraction of nutrients, acoustic cavitation occurs, i.e., the cycle of formation, growth and collapse of microbubbles formed by the propagation of the waves. After the cavitation bubbles collapse, a large amount of energy is released into the micro region close to the surface of the solid phase, thereby leading to the extraction of the analyte and, in some cases, dissolution of the solid material [8]. An analysis of the graphics in Fig. 1 indicates that after 5 cycles of 10 s of ultrasonic shaking, the absorbance signals measured by FAAS or UV–vis remained constant and the temperature measured in the solid–liquid system was 54 °C. As the temperature of the extraction solution increases over prolonged sonication times, a range about 50 °C normally favors the extraction efficiency by increasing the number of acoustic cavitation nuclei formed close to the surface of the solid phase of the suspension (solid–liquid system) [28].

However, when the temperature of the extraction medium approaches the temperature of ebullition of the liquid phase, the sonication efficiency declines due to decreased surface tension of the medium, and increased vapor pressure inside the microbubbles, which causes a reduction in the shock waves [29]. Thus, the sonication time of 5 cycles of 10 s achieved was considered effective in the elements extraction process and besides that this lower sonication time increased the sampling frequency, improving the analytical characteristics of the proposed method.

**Fig. 2:**
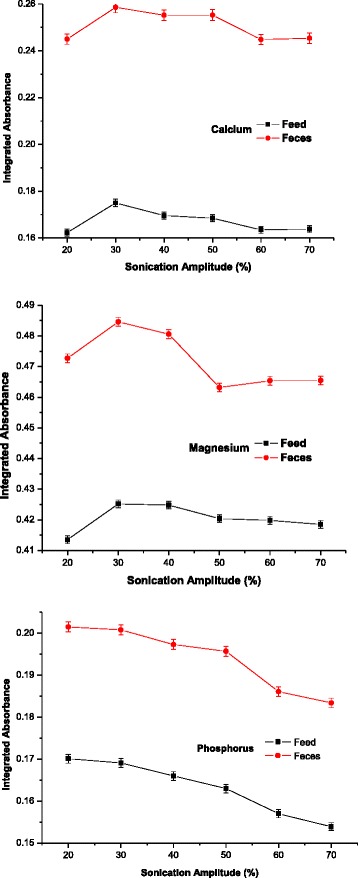
Influence of sonication power on the process of calcium, magnesium and phosphorus extraction from samples of piglet feed and feces. Experimental conditions: sonication time – 5 cycles of 10 s; extracting solution hydrochloric acid 0.10 mol/L

The graphics in Fig. 2 illustrate the influence of the ultrasound amplitude on a scale of 20–70 % of the total work power (340 W). The extraction efficiency of the analytes under study increased as the amplitude rose from 68 to 102 W (20–30 %), remaining constant at the higher amplitudes (105 to 238 W). Since the intensity of the ultrasound transmitted to the medium is directly related to the amplitude of vibration at the tip of the sonotrode, a higher power usually favors an increase in the chemical effects caused by sonication.

However, when very high vibrational amplitude is applied, a large number of cavitation bubbles are generated in the solution, which may be detrimental to the growth and collapse of these bubbles, and leads to a reduction of the energy released through the liquid phase [29–31]. The results indicated that the highest extraction efficiency was achieved on 102 W of the sonication power, because this power caused a lower increase in the temperature of the extracting solution.

The presence of an acidic medium is an essential requirement for quantitative extraction to be attained. For analytical techniques, hydrochloric acid is recommended and it was used in this study because it does not form volatile compounds with analytes, which are the origin of interferences and, in addition, it combined with the ultrasonic action promotes matrix oxidation so that analyte extraction is facilitated [7].

After optimizing the physicochemical parameters (sonication time and power), the accuracy and precision of the extraction method were tested by means of assay to recover the analytes from samples, which content were previously determined by acid mineralization.

The accuracy and precision of the proposed method were also ascertained using the standard reference bovine muscle: NIST RM 8414—National Institute of Standards and Technology. The results obtained from these determinations are shown in Table 2. The QL of calcium, magnesium and phosphorus were 53, 62 and 70 μg/L, while the MQL were 10.6, 12.4 and 14 mg/kg, respectively. The values achieved were sufficiently low to allow the determination of these elements in piglet feed and feces. All of the obtained values presented relative standard deviations of less than 5 %, demonstrating the good repeatability and therefore, demonstrating that the proposed method is precise and that its application in nutrient digestibility studies of animal nutrition is viable.

**Table 2:** Results obtained from the determinations of calcium, magnesium and phosphorus in standard material (NIST RM 8414)

Elements	Certified Values, g/kg	Determined Values, g/kg	
		Sonicated	Mineralized
Calcium	0.145 ± 0.02	0.164 ± 0.01	0.145 ± 0.02
Magnesium	0.960 ± 0.09	1.023 ± 0.03	0.871 ± 0.01
Phosphorus	0.840 ± 0.05	0.830 ± 0.02	0.820 ± 0.03

Results expressed as mean value ± standard deviation (*n* = 4) *P* > 0.05

The comparison of the values of ADC of the Ca and Mg in the three feed used shown in Table 3 indicated that the values determined by the ultrasound-assisted extraction method were in agreement with the values obtained by acid mineralization of the samples. The results presented in Table 3 show that no statistical differences were observed (*P < 0.05*), indicating that the UAE is applicable for this type of sample. Although the high relative standard deviation has been observed in Mg digestibility results, these values were related to differences on the growth performance of the three piglets initially blocked in the same pen, which were submitted to an *ad libitum* feeding system and had different feed intake [22, 26, 32].

**Table 3:** Apparent digestibility coefficient of nutrients in piglet pre-starter diets

Elements	Sonicated			Mineralized		
	Feed_1_	Feed_2_	Feed_3_	Feed_1_	Feed_2_	Feed_3_
Calcium	55.23 ± 6.89	49.83 ± 10.13	51.61 ± 9.23	56.28 ± 5.69	51.92 ± 6.54	51.79 ± 7.18
Magnesium	30.56 ± 12.26	30.10 ± 7.30	21.99 ± 11.47	30.10 ± 8.28	29.14 ± 4.29	20.03 ± 7.83
Phosphorus	64.82 ± 2.68^a^	63.14 ± 2.50^a^	68.16 ± 2.98^a^	45.34 ± 3.01^b^	55.90 ± 1.90^b^	46.52 ± 4.31^b^

Results expressed as mean value ± standard deviation (*n* = 4)

Mean values in the same row followed by different uppercase letters were significantly different by Tukey’s test (*P* < 0.05)

The comparison of the values of ADC of phosphorus in the three feed presented in Table 3 proved to be higher than those calculated using acid mineralization (*P < 0.05*). Since pigs do not synthesize the endogenous phytase which promotes the hydrolysis of the ester linkage between phosphate group and inositol ring, releasing the phosphorus from the phytic acid molecule (inositol hexaphosphate) and considering that ultrasound-assisted extraction method does not mineralize this phytic molecule, the values of ADC calculated using this method were higher, might be due to N_feces_/N_feed_ ratio only consider the phosphorus bioavailable fraction (i.e., fractions extracted in dihydrogen phosphate form). Thus, the ultrasound extraction allowed obtaining digestibility values of phosphorus more consistent to the real phosphorus absorption by piglets [9–13, 33].

## Conclusions

The proposed method for determination of calcium, magnesium and phosphorus in piglet feed using ultrasound-assisted extraction allowed the calculation of the ADC of these nutrients and proved to be equivalent (*P > 0.05*) to those calculated using acid mineralization of the samples. In addition to this finding, the UAE validated method of Ca, Mg and P offered a QL of 53, 62 and 70 μg/L and a MQL of 10.6, 12.4 and 14 mg/kg respectively.

The efficiency of the extraction procedure based on the acoustic cavitation allowed the substitution of hazardous and high handling methods, as the nitric-perchloric acid digestion, by a green chemistry-type procedure. Furthermore, the toxic residues generated in acid extracts after acid mineralization of samples are not generated in the UAE process, thereby contributing to a clean chemistry-type procedure.

The speed of the process and reduced amount of sample required for the determination of these nutrients is useful in animal nutrition and analysis because it is a simple, fast, safe, precise and economical methodology.

## References

[1] Lewis AJ, Southern LL (2001). Swine Nutrition.

[2] Mavromichalis I (2006). Applied nutrition for young pigs.

[3] Kane EA, Jacobson WC, Damewood PM (1959). Use of radioactive chromium oxide in digestibility determinations. J Dairy Sci.

[4] Hanley F (1987). The digestibility of foodstuffs and the effects of feeding selectivity on digestibility determinations in tilapia (*Oreochromis niloticus*). Aquaculture.

[5] Kozloski GV, Flores EMM, Martins AF (1998). Use of chromium oxide in digestibility studies: variation of the results as a function of the measurement method. J Sci Food Agric.

[6] Silva FA, Neves RCF, Quintero-Pinto LG, Padilha CCF, Jorge SMA, Barros MM, Pezzato LE, Padilha PM (2007). Determination of selenium by GFAAS in slurries of fish feces to estimate the bioavailability of this micronutrient in feed used in psiculture. Chemosphere.

[7] Mason TJ, Lorimer JP (1988). Sonochemistry: Theory, Applications and Uses of Ultrasound in Chemistry.

[8] Elik A (2007). Ultrasonic-assisted leaching of trace metals from sediments as a function of pH. Talanta.

[9] Saha DC, Gilbreath RL (1991). Analytical recovery of chromium from diet and feces determined by colorimetry and atomic absorption spectrophotometry. J Sci Food Agric.

[10] Saha DC, Gilbreath RL (1991). Faecal recovery and diurnal variation in excretion of dietary chromium by mature swine. J Sci Food Agric.

[11] Bakker GCM, Jongbloed AW (1994). The effect of housing system on apparent digestibility in pigs, using the classical and marker (chromic oxide, acid-insoluble ash) techniques, in relation to dietary composition. J Sci Food Agric.

[12] Luque-García JL, Luque de Castro MD (2003). Ultrasound: a powerful tool for leaching. Trends Anal Chem.

[13] Hauptli L (2009). Maltodextrina e óleos como fontes de energia para leitões, Ph. D. Thesis.

[14] Rostagno HS, Teixeira A, Donzele JL, Gomes PC, Oliveira RFM, Lopes DC, Ferreira AJP, Toledo Barreto SL (2005). Brazilian Tables for Poultry and Swine: composition of feedstuffs and nutritional requirements.

[15] Shahat TM (1993). Digestibility determination in catfish fingerling using internal and external markers. Vet Med J Giza.

[16] Rosa CR, Moraes M, Neto JAG, Nóbrega JA, Nogueira ARA (2002). Effect of modifiers on thermal behaviour of Se in acid digestates and slurries of vegetables by graphite atomic absorption spectrometry. Food Chem.

[17] Neves RCF, Moraes PM, Saleh MAD, Loureiro VR, Silva FA, Barros MM, Padilha CCF, Padilha PM (2009). FAAS determination of metal nutrients in fish feed after ultrasound extraction. Food Chem.

[18] Currie LA (1999). Nomenclature in evaluation of analytical methods including detection and quantification capabilities (IUPAC Recommendations 1995). Anal Chim Acta.

[19] Manutsewee N, Aeungmaitrepirom W, Varanusupakul P, Imyim A (2007). Determination of Cd, Cu and Zn in fish and mussel by AAS after ultrasound-assisted acid leaching extraction. Food Chem.

[20] Welz B (1992). Symbols and units for integrated absorbance in electrothermal atomic absorption spectrometry (ET-AAS). Spectrochim Acta B At Spectrosc.

[21] Capelo JL, Filgueiras AV, Lavilla I, Bendicho C (1999). Solid–liquid extraction of copper from slurried samples using high intensity probe sonication for electrothermal atomic absorption spectrometry. Talanta.

[22] Cal-Prieto MJ, Felipe-Sotelo M, Carlosena A, Andrade JM, López-Mahía P, Muniategui S, Prada D (2002). Slurry sampling for direct analysis of solid materials by electrothermal atomic absorption spectrometry (ETAAS). A literature review from 1990 to 2000. Talanta.

[23] Dolinsek F, Stupar J, Vrscaj V (1991). Direct determination of cadmium and lead in geological and plant materials by electrothermal atomic absorption spectrometry. J Anal At Spectrom.

[24] Mierzwa J, Dobrowolski R (1994). Silica gel analysis by slurry-sampling graphite-furnace atomic absorption spectrometry. Fresenius J Anal Chem.

[25] Mierzwa J, Dhindsa HS (1998). Determination of arsenic, chromium, and nickel in slurried talc samples by electrothermal AAS. At Spectrosc.

[26] Saleh MAD, Berto DA, Padilha PM (2013). Ultrasound-assisted extraction of Na and K from swine feed and its application in a digestibility assay: A green analytical procedure. Ultrason Sonochem.

[27] SAS Institute Inc (1999). SAS OnlineDoc®, version 8, Cary.

[28] Filgueiras AV, Capelo JL, Lavilla I, Bendicho C (2000). Comparison of ultrasound-assisted extraction and microwave-assisted digestion for determination of magnesium, manganese and zinc in plant samples by flame atomic absorption spectrometry. Talanta.

[29] Elik A (2005). Ultrasound assisted pseudo-digestion of street dust samples prior to determination by atomic absorption spectrometry. Talanta.

[30] Nascentes CC, Korn M, Arruda MAZ (2001). A fast ultrasound-assisted extraction of Ca, Mg, Mn and Zn from vegetables. Microchem J.

[31] Ruiz-Jiménez J, Luque-Garcia JL, Luque de Castro MD (2003). Dynamic ultrasound-assisted extraction of cadmium and lead from plants prior to electrothermal atomic absorption spectrometry. Anal Chim Acta.

[32] Bermejo-Barrera P, Moreda-Piñeiro A, Moreda-Piñeiro J, Bermejo-Barrera A (1998). Comparative study on the use of Ir, W and Zr-coated graphite tubes for the determination of chromium in slurries of human scalp hair by electrothermal atomic absorption spectrometry. Fresenius J Anal Chem.

[33] Moraes PM, Loureiro VR, Padilha PM, Neves RCF, Saleh MAD, Santos FA, Silva FA (2009). Determinação de fósforo biodisponível em rações de peixes utilizando extração assistida por ultra-som e espectrofotometria no visível. Quim Nova.

